# Mean versus variability of lipid measurements over 6 years and incident cardiovascular events: More than a decade follow-up

**DOI:** 10.3389/fcvm.2022.1065528

**Published:** 2022-12-09

**Authors:** Soroush Masrouri, Leila Cheraghi, Niloofar Deravi, Neda Cheraghloo, Maryam Tohidi, Fereidoun Azizi, Farzad Hadaegh

**Affiliations:** ^1^Prevention of Metabolic Disorders Research Center, Research Institute for Endocrine Sciences, Shahid Beheshti University of Medical Sciences, Tehran, Iran; ^2^Department of Epidemiology and Biostatistics, Research Institute for Endocrine Sciences, Shahid Beheshti University of Medical Sciences, Tehran, Iran; ^3^Department of Epidemiology and Biostatistics, School of Public Health, Tehran University of Medical Sciences, Tehran, Iran; ^4^Endocrine Research Center, Research Institute for Endocrine Sciences, Shahid Beheshti University of Medical Sciences, Tehran, Iran

**Keywords:** lipid variability, lipid mean levels, cardiovascular diseases, Tehran Lipid and Glucose Study, prospective cohort study

## Abstract

**Background:**

Lipid variability (LV) has emerged as a contributor to the incidence of cardiovascular diseases (CVD), even after considering the effect of mean lipid levels. However, these associations have not been examined among people in the Middle East and North Africa (MENA) region. We aimed to investigate the association of 6-year mean lipid levels versus lipid variability with the risk of CVD among an Iranian population.

**Methods:**

A total of 3,700 Iranian adults aged ≥ 30 years, with 3 lipid profile measurements, were followed up for incident CVD until March 2018. Lipid variability was measured as standard deviation (SD), coefficient of variation (CV), average real variability (ARV), and variability independent of mean (VIM). The effects of mean lipid levels and LV on CVD risk were assessed using multivariate Cox proportional hazard models.

**Results:**

During a median 14.5-year follow-up, 349 cases of CVD were recorded. Each 1-SD increase in the mean levels of total cholesterol (TC), low-density lipoprotein cholesterol (LDL-C), TC/high-density lipoprotein cholesterol (HDL-C), and non-HDL-C increased the risk of CVD by about 26–29%; for HDL-C, the risk was significantly lower by 12% (all *p*-values < 0.05); these associations resisted after adjustment for their different LV indices. Considering LV, each 1-SD increment in SD and ARV variability indices for TC and TC/HDL-C increased the risk of CVD by about 10%; however, these associations reached null after further adjustment for their mean values. The effect of TC/HDL-C variability (measured as SD) and mean lipid levels, except for LDL-C, on CVD risk was generally more pronounced in the non-elderly population.

**Conclusion:**

Six-year mean lipid levels were associated with an increased future risk of incident CVD, whereas LV were not. Our findings highlight the importance of achieving normal lipid levels over time, but not necessarily consistent, for averting adverse clinical outcomes.

## Introduction

Cardiovascular diseases (CVD), one of the most common public health issues in the world and especially in the Middle East and North Africa (MENA) region, has high morbidity and mortality (1, 2). Managing CVD risk factors in the general population is considered an ongoing challenge for the decision-makers in the primary healthcare system (3). Despite the favorable trends for different lipid measures among the Iranian population over the last decade (4, 5), the prevalence of dyslipidemia is still high (6). According to national data from 2016, among more than 21,000 adults aged over 25 years, about 80% of the individuals had at least one lipid abnormality [i.e., about 70, 40, 28, and 27% had low high-density lipoprotein cholesterol (HDL-C), high non-HDL-C, hypertriglyceridemia, and hypercholesterolemia, respectively] (7). During 10 years of follow-up among the Tehranian population, we demonstrated that about 30% of the population attributable fraction of CVD was related to hypercholesterolemia and low HDL-C (6). In recent years, variability in lipid measures has been shown to be associated with unfavorable cardiovascular outcomes in some but not all studies (8). A recent systematic review and meta-analysis, including six cohort studies mainly conducted among the East Asian population, found that higher variability of total cholesterol (TC), low-density lipoprotein cholesterol (LDL-C), and HDL-C but not triglycerides (TG) were associated with incident CVD events (9). Importantly, significant heterogenicity was found among the included studies, and the association between lipid variability (LV) and CVD events became weaker after further adjustment for the mean level of the lipid measure of interest. Recently, another cohort study among an East Asian population found that mildly abnormal lipid levels but not their variability significantly contributed to the occurrence of CVD events (8).

To our best knowledge, this study is the first to examine the association between longitudinal lipid measures and incident CVD events in the MENA, a region with a high burden of dyslipidemia. In the current study, we compared the impact of LV versus mean levels of different lipid measures on incident CVD events in the population-based cohort of the Tehran Lipid and Glucose Study (TLGS) with more than a decade of follow-up.

## Materials and methods

### Study design and sample

A prospective study was conducted among the participants of the TLGS, an ongoing large-scale community-based cohort study, which was originally established to estimate the prevalence and incidence of non-communicable diseases (NCDs) and to prevent NCDs by advancing a healthier lifestyle among residents of district 13 of Tehran. Enrolment of the study population was carried out in two phases, including the first (1999–2001; *n* = 15,005) and the second (2002–2005; *n* = 3550). So far, participants of the TLGS have been followed up for 20 years in tri-annual intervals. We obtained written consent from all subjects after being informed regarding the general aspects of the study and its methods; the study was also approved by the Ethical Committee of the Research Institute for Endocrine Sciences (RIES). Additional details about the study design and the characteristics of the study population of TLGS have been reported previously (10).

### Study population

For the current study, 7,123 participants aged ≥ 30 years who underwent a baseline examination in 2002–2005 were included; participants underwent lipid measurements in 3 phases including 2nd phase: 2002–2005 (considered as the baseline in the current study), 3rd phase: 2005–2008, and 4th phase: 2009–2011; the interval between these phases was about 3 years. Of 7,123 participants, we excluded those who had a previous history of CVD or developed CVD during the measurement period (*n* = 1027) and those who did not attend the second and/or third examinations during the measurement period (*n* = 1693). Considering overlap features, we further excluded participants with missing data regarding lipid measures (*n* = 126) or covariates (*n* = 577) during the measurement period; ultimately, the study population included 3,700 subjects who were followed up for incident CVD.

### Clinical and laboratory measurements

Interviewer-administered standard questionnaires were used to collect demographic information, past medical history of CVD, family history of premature CVD (FH-CVD), drug history, and smoking status from subjects during the visits. Body mass index (BMI, kg/m^2^) was calculated as body weight divided by height squared. After resting for 15 min in a sitting position, blood pressure was measured by trained personnel and on the right arm with a standard mercury sphygmomanometer; the systolic and diastolic blood pressures (SBP and DBP, respectively) were calculated as the average of two measures. Blood samples for biochemical analysis were collected between 7:00 and 9:00 a.m., after a 12–14 h overnight fasting, including fasting plasma glucose (FPG) and lipid measures (i.e., TC, TG, and HDL-C). For the oral glucose tolerance test (OGTT), in participants without known diabetes, 82.5 g oral glucose monohydrate solution was administered orally (equivalent to 75 g anhydrous glucose), and blood samples were taken after 2 h (2-h post-challenge glucose, 2 h-PG). Analysis of the blood samples from all subjects was performed on the same day of blood collection in the TLGS research laboratory. TC and TG were assayed using an enzymatic colorimetric method with cholesterol esterase-cholesterol oxidase and glycerol phosphate oxidase, respectively. HDL-C was assessed following precipitation of the apolipoprotein B-containing lipoproteins with phosphotungstic acid. In baseline and follow-up assays, intra- and inter-assay coefficients of variation (CVs) were 0.5 and 2%, respectively, for both TC and HDL-C. Intra- and inter-assay CV were 0.6 and 1.6% for TG, respectively. All of these CV% were less than the maximum allowable imprecision for lipid measurements and had little effects on total lipid variability (11, 12). A modified Friedewald formula was used to calculate LDL-C (13). Non-HDL-C was calculated by subtracting HDL-C from TC. TC/HDL-C and TG/HDL-C were calculated as TC and TG divided by HDL-C, respectively. The analyses were performed by Selectra 2 auto-analyzer (Vital Scientific, Spankeren, Netherlands) and Pars Azmon kits (Pars Azmon Inc., Tehran, Iran). Analyses of samples were carried out only as the internal quality control met acceptable criteria.

### Definition of terms

Mean and different variability indices, including standard deviation (SD), CV, variability independent of the mean (VIM), and average real variability (ARV) for each lipid parameter, including TC, TG, HDL-C, LDL-C, TC/HDL-C, TG/HDL-C, and non-HDL-C were estimated using values measured during the three tri-annual consecutive examinations. VIM was calculated as 100 × SD/mean^β^, where β is the regression coefficient for the natural logarithm of SD on the mean (Ln SD/Ln mean). ARV was calculated according to the following formula, where N is the number of lipid values corresponding to a given participant:


ARV=1N-1Σi-1N-1|Valuei-+1Value|i

In our study, we divided our participants’ smoking status into two categories, including current smokers versus ex-/never-smokers. FH-CVD was defined as having a previous diagnosis of myocardial infarction (MI), stroke, or sudden cardiac death (SCD) among female first-degree relatives aged < 65 years (male < 55 years). Prevalent CVD was any self-report history of MI, ischemic heart disease, CCU admission, angiography proven coronary heart disease (CHD) [defined as > 50% stenosis in at least one of the major coronary vessels (14–16)], angioplasty, coronary artery bypass grafting, and history of cerebrovascular accidents. Diabetes was defined as one of the following criteria: (a) FPG ≥ 7.0 mmol/L (126 mg/dL); (b) 2-h PG ≥ 11.1 mmol/L (200 mg/dL); (c) using glucose-lowering medications.

The primary outcome for the present study was any occurrence of the first CVD. Details of the definitions and outcomes analysis are reported elsewhere (10, 17). In summary, a trained nurse followed subjects each year regarding any cardiovascular outcomes that occurred over the previous year *via* phone call interviews till March 2018. Then, a trained physician determined the diagnosis of the events *via* collecting complementary data from medical documents or death certificates, in case of mortality, during a home or hospital visit. All the collected documents underwent an evaluation administered by an outcome committee from TLGS, including an internist, endocrinologist, cardiologist, pathologist, epidemiologist, and other experts, as deemed necessary, and the final diagnosis was based on the majority consensus of the committee members. In the present study, a CVD event included the following:

• angiographic-proven CHD (defined as > 50% stenosis in at least one of the major coronary vessels);
• fatal and non-fatal stroke;
• unstable angina pectoris;
• probable fatal and non-fatal MI;
• definite fatal and non-fatal MI;
• SCD (death within less than 1 h of symptom onset when witnessed or within 24 h if the individual was last observed to be alive).

### Statistical analysis

Summary statistics for the study population are expressed as mean ± SD values and frequencies (%) for continuous and categorical variables, respectively. Comparison of the baseline characteristics of the participants stratified by the quartiles of TC variability (using SD) and occurrence of outcome (CVD) was performed using the ANOVA test or Student’s *t*-test for continuous and the Pearson’s χ^2^ test for categorical variables, as appropriate.

After confirming that the proportionality assumptions were appropriate (using the Schoenfeld’s global test of residuals), Cox proportional hazards regression was implemented to estimate adjusted hazard ratios (HRs) and 95% CIs in relation to subsequent risk of CVD for the mean lipid levels and indices of LV (SD, CV, ARV, and VIM). Mean lipid levels and indices of LV were examined as both continuous (per each standard deviation increase for individual and combined lipid parameters) and categorical variables, considering the first quartile as reference. All models were adjusted for smoking status, age, sex, FH-CVD (at baseline), as well as using lipid-lowering and anti-hypertensive medications, diabetes, and average values of BMI and SBP (during the measurement period). In model 2, the analysis for each lipid parameter was further adjusted for the mean value of lipid measure of interest (i.e., for the analysis of TC, adjusted for mean level of TC). Generally, correlations between predictors, especially LV and mean lipid values, may cause multicollinearity issues; we assessed the presence of multicollinearity by calculating the variation inflation factor (VIF) statistic in the regression models. Considering the VIF of less than 5, no multicollinearity was found between variables in different models. Accordingly, participants without prevalent CVD were followed from baseline until the occurrence of a new cardiovascular event (the exact date of which was considered as the date of the end point event). Censoring was defined as loss to follow-up, non-CVD death, or the end of study (March 2018).

To explore differences in the effect of the mean lipid levels or LV on CVD in different population characteristics, after assessment of interactions, subgroup analyses by age group (< 60, ≥ 60 years), sex, obesity status (BMI < 30 and ≥ 30 kg/m^2^), smoking status, diabetes, hypertension, and the use of lipid-lowering medications were carried out for the top quartile (Q4) versus the first to third quartiles (Q1–Q3), in separate models. For the interaction *p*-value in the subgroup analysis, Bonferroni correction was not considered due to the relatively small number of cases since using it might have increased the likelihood of type 2 errors.

To address the issue of selection bias due to missing data, we performed multiple imputations as a sensitivity analysis; multivariate imputation based on the twofold Fully Conditional Specification (FCS) algorithm method for longitudinal records of routinely collected clinical data was utilized to impute missing data for fixed and time-varying variables (18, 19). Twofold FCS accounts for the longitudinal structure of the data by imputing each variable, restricting its imputation to time blocks, by using only data from that particular time point and the immediately adjacent ones, which prevents collinearity issues and over-fitting (20–22). The imputation model included all variables in the analysis plus the outcome and survival-time as explanatory variables; we also included creatinine as an auxiliary variable (variables in the data that are not included in the analysis but are correlated to the variables of interest or help keep the missing process random) (23). The number of imputations was set based on at least 1 imputation per percent of incomplete cases (24, 25). Our study sample had ∼35% incomplete cases; therefore, we generated 35 imputed datasets (with 20 among time and 5 within time iterations) according to multiple imputation procedures. Finally, results from 35 Cox regression analyses were combined according to Rubin’s Rule (26). All statistical analyses were performed using SPSS for windows version 20 and STATA version 14 SE (StataCorp LP, TX, USA). Two-tailed *P*-values below < 0.05 indicates statistically significant.

## Results

### Baseline characteristics

The study population included 3,700 participants (women = 2,157) with a mean age (SD) of 46.59 (11.39) years. Table 1 summarizes the characteristics of the TLGS participants classified by quartiles of SD-TC. The mean ± SD values of average TC in the Q1–Q4 groups were (4.89 ± 0.86), (5.00 ± 0.83), (5.13 ± 0.84), and (5.50 ± 0.92) mmol/L, respectively. Compared to those in lower SD-TC quartiles, subjects in higher quartiles had higher levels of TC in all three examinations; they also were generally older, less likely to be men, had higher baseline levels of SBP, FPG, 2-h PG, co-morbid conditions including diabetes FH-CVD, and the use of lipid-lowering and antihypertensive agents; however, no significant difference was observed regarding current smoking and baseline BMI.

**TABLE 1:** Characteristics of participants according to the quartiles of total cholesterol variability (measured as standard deviation): Tehran Lipid and Glucose Study.

	Total	Q1 (<0.24) (mmol/L)	Q2 (0.24–0.40) (mmol/L)	Q3 (0.40–0.60) (mmol/L)	Q4 (≥0.60) (mmol/L)	*P*-value
Number of participants	3700	914	939	919	928	
**Continuous variables, mean ± SD**						
Age (year)	46.59 ± 11.39	45.89 ± 11.53	46.43 ± 11.63	46.31 ± 11.47	47.70 ± 10.85	< 0.01
SBP (mmHg)	117.11 ± 17.53	116.17 ± 16.17	117.02 ± 17.42	116.79 ± 18.14	118.43 ± 18.25	0.04
BMI (kg/m^2^)	28.11 ± 4.45	27.96 ± 4.23	28.08 ± 4.39	27.96 ± 4.43	28.43 ± 4.71	0.08
FPG (mmol/L)	5.48 ± 1.63	5.32 ± 1.39	5.34 ± 1.23	5.50 ± 1.63	5.77 ± 2.10	< 0.01
2-h PG (mmol/L)	6.70 ± 3.01	6.44 ± 2.69	6.57 ± 2.65	6.60 ± 3.12	7.21 ±3.50	< 0.01
TC at first exam (mmol/L)	5.13 ± 1.02	4.88 ± 0.87	4.98 ± 0.88	5.09 ± 0.95	5.58 ± 1.21	< 0.01
TC at second exam (mmol/L)	5.11 ± 0.99	4.88 ± 0.86	4.98 ± 0.86	5.08 ± 0.92	5.49 ± 1.17	< 0.01
TC at third exam (mmol/L)	5.15 ± 1.00	4.91 ± 0.87	5.07 ± 0.86	5.21 ± 0.90	5.42 ± 1.25	< 0.01
**Categorical variables, number (%)**						
Men	1543 (41.7)	414 (45.30)	438 (46.65)	386 (42.00)	305 (32.87)	< 0.01
Current smoking	428 (11.6)	100 (10.94)	121 (12.89)	113 (12.30)	94 (10.13)	0.23
FH-CVD	375 (10.1)	86 (9.41)	89 (9.48)	83 (9.03)	117 (12.61)	0.04
Lipid-lowering drugs	466 (12.6)	49 (5.36)	53 (5.64)	83 (9.03)	281 (30.28)	< 0.01
Anti-hypertensive drugs	614 (16.6)	115 (12.58)	134 (14.27)	149 (16.21)	216 (23.28)	< 0.01
Diabetes	718 (19.4)	132 (14.44)	159 (16.93)	163 (17.74)	264 (28.45)	< 0.01

SBP, systolic blood pressure; BMI, body mass index; FPG, fasting plasma glucose; 2-h PG, 2 h post-challenge glucose; TC, total cholesterol; FH-CVD, family history of CVD.

The characteristics of individuals according to the occurrence of outcome are presented in Supplementary Table 1. Compared to those without incident CVD, subjects who developed CVD were more likely to be men and generally had a worse cardiometabolic profile; furthermore, there was no significant difference regarding smoking status.

### Association of mean lipid levels with cardiovascular diseases

**FIGURE 1:**
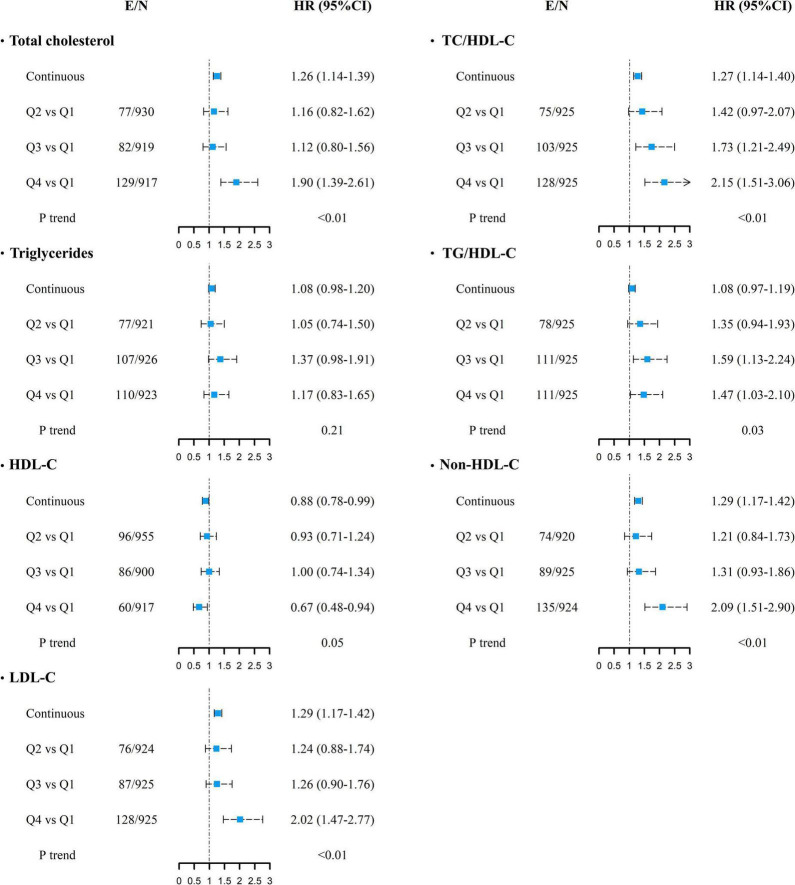
Multivariable-adjusted HRs (95% CI) for incident cardiovascular diseases associated with mean lipid levels of TC, TG, HDL-C, LDL-C, TC/HDL-C, TG/HDL-C, and non-HDL-C. Adjusted for age, sex, smoking status, diabetes mellitus, family history of CVD, use of anti-hypertensive drugs, lipid-lowering drugs, and average body mass index and systolic blood pressure. *P* for trend was calculated across quartiles using multivariable Cox regression model. Continuous variables are shown for each 1-SD increase. E/N, number of events per number of subjects in each category; HR, hazard ratio; CI, confidence interval; TC, total cholesterol; TG, triglycerides; HDL-C, high-density lipoprotein cholesterol; LDL-C, low-density lipoprotein cholesterol. For all lipid parameters, first quartile (Q1) is the reference category.

During a median of 14.5 years of follow-up (interquartile range: 13.8–15.5 years) after the first examination, 349 cases of CVD were recorded. HRs and their 95% CI for the risk of CVD according to the mean levels of the individual (TC, TG, HDL-C, and LDL-C) and combined lipid parameters (TC/HDL-C, TG/HDL-C, and non-HDL-C) are shown in Figure 1. After adjusting for age, sex, smoking, family history of premature CVD, average BMI and SBP levels, use of antihypertensive and lipid-lowering agents, and diabetes, each 1-SD increase in the mean levels of TC, LDL-C, TC/HDL-C, and non-HDL-C increased the risk of CVD by about 26–29%; for HDL-C, the risk was significantly lower by 12% (all *p*-values < 0.05). Additionally, the risk of CVD was significantly higher in the top quartile of mean TC, LDL-C, and non-HDL-C levels versus the lowest one. The third and top quartiles of mean TC/HDL-C and TG/HDL-C also showed significantly higher risk and the top quartile of mean HDL-C reduced the risk of CVD by 33%. Increasing values in the mean levels of TC, LDL-C, non-HDL-C, TC/HDL-C, and TG/HDL-C were associated with a higher risk of CVD in the multivariable-adjusted models; regarding HDL-C, however, a significant inverse trend was observed (all *p* for trends < 0.05). The results were generally similar after adjustment with variability indices; however, the association of 1-SD increase in the mean of HDL-C reached the non-significant level after further adjustment for SD- or ARV-HDL-C. Furthermore, the association between mean TG/HDL-C and incident CVD became significant after adjustment for ARV-TG/HDL-C (data not shown).

### Association of lipid variability with cardiovascular diseases

As shown in Tables 2, 3, in the presence of all confounders except for mean value of lipid measures (model 1), each 1-SD increment in ARV-TC and ARV-TC/HDL-C significantly increased the risk of CVD [HR (95% CI); 1.11 (1.00–1.23) and 1.11 (1.02–1.22), respectively]. The associations of SD-TC and SD-TC/HDL-C with the risk of CVD events were similar to those of the ARV indices and conferred about a 10% increased risk; however, with a borderline significance for SD-TC (*p*-value = 0.055). Regarding the lipid parameters as categorized variables, compared to the reference quartile, the top quartile of CV-LDL-C and the third quartile of ARV-TG conferred 29% lower and 41% higher risk of CVD in model 1, respectively; moreover, moving from the first to fourth quartile of LDL-C variability applying SD, CV, and ARV tended to be associated with a lower risk of CVD events (all *p* for trends < 0.06). All of these significant associations between LV and CVD events using different lipid parameters reached null after further adjustment for the mean lipid level of each lipid parameter. Moreover, the third quartile of VIM-TG/HDL-C versus the first quartile had a significant association with a lower risk of CVD in both models 1 and 2 [HR (95% CI); 0.74 (0.55–1.00)].

**TABLE 2:** Hazard ratios and 95% CI of incident CVD for TC, TG, HDL-C, and LDL-C variability: Tehran Lipid and Glucose Study.

	SD		CV		ARV		VIM	
	Model 1	Model 2	Model 1	Model 2	Model 1	Model 2	Model 1	Model 2
**TC**								
Q_1_	1 (reference)	1 (reference)	1 (reference)	1 (reference)	1 (reference)	1 (reference)	1 (reference)	1 (reference)
Q_2_	0.88 (0.64–1.21)	0.85 (0.62–1.17)	0.84 (0.62–1.14)	0.83 (0.62–1.13)	1.00 (0.73–1.36)	0.95 (0.70–1.30)	0.78 (0.58–1.07)	0.77 (0.57–1.05)
Q_3_	1.06 (0.78–1.44)	0.99 (0.73–1.35)	0.92 (0.68–1.24)	0.91 (0.67–1.22)	1.00 (0.73–1.37)	0.92 (0.67–1.27)	0.90 (0.67–1.21)	0.89 (0.66–1.20)
Q_4_	1.12 (0.82–1.53)	0.98 (0.72–1.35)	0.95 (0.70–1.29)	0.96 (0.71–1.30)	1.20 (0.88–1.64)	1.06 (0.77–1.46)	0.93 (0.69–1.26)	0.95 (0.70–1.28)
Per 1-SD increase	1.11 (1.00–1.22)*	1.05 (0.94–1.16)	1.06 (0.96–1.19)	1.08 (0.98–1.20)	1.11 (1.00–1.23)*	1.05 (0.95–1.17)	1.07 (0.96–1.19)	1.08 (0.98–1.20)
*P* for trend	0.29	0.83	0.84	0.91	0.26	0.73	0.82	0.92
**TG**								
Q_1_	1 (reference)	1 (reference)	1 (reference)	1 (reference)	1 (reference)	1 (reference)	1 (reference)	1 (reference)
Q_2_	1.06 (0.77–1.46)	1.05 (0.76–1.45)	1.04 (0.76–1.41)	1.04 (0.76–1.41)	1.12 (0.81–1.54)	1.09 (0.79–1.51)	0.99 (0.74–1.33)	0.99 (0.74–1.33)
Q_3_	1.14 (0.83–1.56)	1.11 (0.80–1.52)	0.95 (0.70–1.29)	0.94 (0.69–1.28)	1.41 (1.04–1.93)*	1.34 (0.98–1.84)	0.85 (0.62–1.15)	0.84 (0.62–1.15)
Q_4_	1.21 (0.89–1.65)	1.10 (0.77–1.57)	1.20 (0.89–1.62)	1.17 (0.86–1.58)	1.10 (0.80–1.51)	0.94 (0.66–1.36)	1.14 (0.86–1.53)	1.13 (0.84–1.51)
Per 1-SD increase	1.05 (0.96–1.15)	0.99 (0.87–1.13)	1.02 (0.92–1.13)	1.00 (0.44–2.23)	1.01 (0.91–1.12)	0.94 (0.82–1.08)	1.00 (0.90–1.12)	0.99 (0.89–1.11)
*P* for trend	0.20	0.54	0.33	0.45	0.36	0.85	0.60	0.65
**HDL-C**								
Q_1_	1 (reference)	1 (reference)	1 (reference)	1 (reference)	1 (reference)	1 (reference)	1 (reference)	1 (reference)
Q_2_	1.05 (0.79–1.39)	1.07 (0.81–1.42)	0.86 (0.64–1.16)	0.85 (0.63–1.15)	1.07 (0.81–1.41)	1.09 (0.82–1.44)	0.94 (0.70–1.26)	0.93 (0.70–1.26)
Q_3_	1.01 (0.74–1.37)	1.04 (0.76–1.43)	1.09 (0.82–1.46)	1.09 (0.81–1.46)	0.97 (0.69–1.36)	1.00 (0.71–1.42)	1.04 (0.78–1.39)	1.04 (0.79–1.39)
Q_4_	0.90 (0.65–1.23)	0.97 (0.70–1.35)	0.95 (0.71–1.27)	0.94 (0.70–1.26)	0.90 (0.66–1.23)	0.98 (0.71–1.36)	0.93 (0.69–1.26)	0.94 (0.69–1.27)
Per 1-SD increase	0.96 (0.86–1.07)	0.99 (0.88–1.12)	1.02 (0.92–1.14)	1.02 (0.92–1.14)	0.92 (0.82–1.03)	0.95 (0.84–1.07)	1.01 (0.91–1.13)	1.01 (0.92–1.13)
*P* for trend	0.45	0.84	0.90	0.95	0.39	0.76	0.83	0.88
**LDL-C**								
Q_1_	1 (reference)	1 (reference)	1 (reference)	1 (reference)	1 (reference)	1 (reference)	1 (reference)	1 (reference)
Q_2_	1.03 (0.76–1.39)	1.02 (0.75–1.38)	0.92 (0.68–1.23)	0.94 (0.70–1.26)	0.98 (0.72–1.34)	0.96 (0.71–1.31)	0.92 (0.68–1.23)	0.94 (0.70–1.26)
Q_3_	0.85 (0.62–1.16)	0.78 (0.57–1.07)	0.77 (0.57–1.05)	0.78 (0.58–1.06)	0.98 (0.72–1.35)	0.92 (0.67–1.26)	0.86 (0.63–1.16)	0.85 (0.63–1.15)
Q_4_	0.91 (0.67–1.25)	0.78 (0.57–1.07)	0.71 (0.51–0.98)*	0.77 (0.55–1.06)	1.07 (0.78–1.47)	0.95 (0.69–1.30)	0.75 (0.55–1.03)	0.78 (0.57–1.06)
Per 1-SD increase	1.03 (0.93–1.15)	0.97 (0.87–1.08)	0.97 (0.87–1.08)	1.00 (0.90–1.12)	1.07 (0.96–1.19)	1.01 (0.91–1.12)	0.98 (0.88–1.09)	0.99 (0.89–1.11)
*P* for trend	0.36	0.05	0.02	0.06	0.66	0.71	0.06	0.09

Model 1: Age, sex, smoking, lipid-lowering drugs, anti-hypertensive drugs, family history of CVD, diabetes, and the average values of BMI and SBP.

Model 2: Model 1 + average lipid levels for each lipid parameter (TC, TG, HDL-C, LDL-C).

HR, hazard ratio; CI, confidence interval; CVD, cardiovascular disease; TC, total cholesterol; TG, triglycerides; HDL-C, high-density lipoprotein cholesterol; LDL-C, low-density lipoprotein cholesterol; BMI, body mass index; SBP, systolic blood pressure; SD, standard deviation; CV, coefficient of variation; ARV, average real variability; VIM, variability independent of mean.

*P* for trend was calculated across quartiles using the multivariable Cox regression model.

*P < 0.05.

**TABLE 3:** Hazard ratios and 95% CI of incident CVD for TC/HDL-C, TG/HDL-C, and non-HDL-C variability: Tehran Lipid and Glucose Study.

	SD		CV		ARV		VIM	
	Model 1	Model 2	Model 1	Model 2	Model 1	Model 2	Model 1	Model 2
**TC/HDL-C**								
Q_1_	1 (reference)	1 (reference)	1 (reference)	1 (reference)	1 (reference)	1 (reference)	1 (reference)	1 (reference)
Q_2_	0.87 (0.63–1.22)	0.83 (0.60–1.16)	0.94 (0.68–1.30)	0.91 (0.66–1.26)	0.88 (0.64–1.22)	0.84 (0.61–1.17)	0.94 (0.70–1.28)	0.93 (0.68–1.25)
Q_3_	0.91 (0.67–1.25)	0.81 (0.59–1.12)	0.97 (0.72–1.31)	0.92 (0.68–1.24)	0.99 (0.73–1.36)	0.88 (0.64–1.21)	0.87 (0.64–1.19)	0.86 (0.63–1.17)
Q_4_	1.21 (0.90–1.63)	0.93 (0.68–1.29)	1.10 (0.81–1.49)	1.03 (0.76–1.40)	1.20 (0.89–1.62)	0.93 (0.67–1.29)	0.98 (0.72–1.32)	0.99 (0.73–1.34)
Per 1-SD increase	1.10 (1.02–1.20)*	1.00 (0.90–1.10)	1.03 (0.93–1.15)	1.00 (0.90–1.11)	1.11 (1.02–1.22)*	1.01 (0.91–1.11)	0.99 (0.89–1.10)	1.00 (0.90–1.10)
*P* for trend	0.15	0.74	0.51	0.82	0.13	0.81	0.79	0.88
**TG/HDL-C**								
Q_1_	1 (reference)	1 (reference)	1 (reference)	1 (reference)	1 (reference)	1 (reference)	1 (reference)	1 (reference)
Q_2_	1.04 (0.75–1.43)	1.02 (0.74–1.42)	1.02 (0.75–1.37)	1.01 (0.75–1.37)	1.00 (0.73–1.37)	0.97 (0.71–1.34)	0.88 (0.66–1.18)	0.88 (0.66–1.18)
Q_3_	0.96 (0.69–1.32)	0.93 (0.66–1.29)	0.81 (0.59–1.11)	0.81 (0.59–1.11)	0.97 (0.70–1.32)	0.91 (0.65–1.25)	0.74 (0.55–1.00)*	0.74 (0.55–1.00)*
Q_4_	1.20 (0.88–1.64)	1.11 (0.76–1.62)	1.13 (0.85–1.52)	1.10 (0.82–1.48)	1.03 (0.76–1.41)	0.87 (0.60–1.27)	0.99 (0.74–1.32)	0.98 (0.74–1.31)
Per 1-SD increase	1.03 (0.94–1.13)	0.95 (0.82–1.10)	0.99 (0.89–1.10)	0.97 (0.87–1.08)	1.00 (0.90–1.11)	0.89 (0.75–1.05)	0.97 (0.87–1.08)	0.96 (0.86–1.07)
*P* for trend	0.30	0.78	0.65	0.82	0.89	0.43	0.67	0.63
**Non-HDL-C**								
Q_1_	1 (reference)	1 (reference)	1 (reference)	1 (reference)	1 (reference)	1 (reference)	1 (reference)	1 (reference)
Q_2_	0.85 (0.62–1.16)	0.83 (0.61–1.14)	0.84 (0.63–1.13)	0.85 (0.63–1.13)	0.83 (0.60–1.14)	0.80 (0.58–1.11)	0.88 (0.65–1.19)	0.88 (0.66–1.19)
Q_3_	0.94 (0.69–1.28)	0.87 (0.64–1.19)	0.86 (0.63–1.17)	0.87 (0.64–1.19)	0.94 (0.69–1.28)	0.87 (0.64–1.18)	0.80 (0.59–1.09)	0.80 (0.59–1.09)
Q_4_	0.96 (0.70–1.31)	0.83 (0.60–1.15)	0.85 (0.62–1.16)	0.90 (0.66–1.23)	1.03 (0.75–1.41)	0.91 (0.66–1.25)	0.88 (0.65–1.20)	0.88 (0.64–1.19)
Per 1-SD increase	1.06 (0.96–1.18)	1.00 (0.90–1.12)	1.01 (0.90–1.12)	1.04 (0.93–1.15)	1.10 (0.99–1.22)	1.03 (0.93–1.15)	1.01 (0.91–1.12)	1.03 (0.93–1.15)
*P* for trend	0.98	0.35	0.34	0.56	0.65	0.73	0.30	0.44

Model 1: Age, sex, smoking, lipid-lowering drugs, anti-hypertensive drugs, family history of CVD, diabetes, and the average values of BMI and SBP.

Model 2: Model 1 + average lipid levels for each lipid parameter (TC/HDL-C, TG/HDL-C, non-HDL-C).

HR, hazard ratio; CI, confidence interval; CVD, cardiovascular disease; TC, total cholesterol; TG, triglycerides; HDL-C, high-density lipoprotein cholesterol; BMI, body mass index; SBP, systolic blood pressure; SD, standard deviation; CV, coefficient of variation; ARV, average real variability; VIM, variability independent of mean.

*P* for trend was calculated across quartiles using the multivariable Cox regression model.

*P < 0.05.

### Subgroup analysis

Subgroup analysis according to age, sex, obesity status, current smoking, co-morbid conditions (diabetes and hypertension), and use of lipid-lowering medications for mean levels and LV of individual and combined lipid parameters (Q4 vs. Q1–3) was carried out (Supplementary Figures 1, 2). Significant interactions of hypertension with the mean levels of TC, use of lipid-lowering medication with LDL-C, and age groups (60 years and older vs. younger) with all lipid indices, except for LDL-C, on the risk of CVD were observed (all *p* for interactions < 0.05).

The Q4 versus the Q1–3 group of mean lipid levels was associated with a significantly higher risk of CVD among those without hypertension for TC and those not on lipid-lowering medication for LDL-C. Notably, compared to the Q1–3 group, the increased risk of CVD associated with the Q4 group of mean TC, TC/HDL-C, and non-HDL-C was more pronounced significantly in those younger than 60 years old. Similarly, the last quartile of HDL-C reduced the risk only among younger participants. In participants 60 years and older higher mean levels of TG and TG/HDL-C, however, significantly decreased the risk of CVD.

In the stratified analysis regarding variability (SD) of lipid parameters, no significant association across subgroups for individual lipid parameters was present. For the combined lipid parameters, the risk of CVD was significantly more pronounced for the high variability of TC/HDL-C among smokers and those aged < 60 years; for TG/HDL-C variability, in participants with BMI ≥ 30 kg/m^2^, significantly higher risk was observed; of note, high non-HDL-C variability (Q4) compared to lower variability status (Q1–3) was associated with better survival among women (*p*-value = 0.06), all *p* for interactions < 0.05.

### Sensitivity analysis

Results of the sensitivity analysis of imputed data are presented in Supplementary Tables 2–4. After imputation of missing data, findings were generally in line with the complete case analysis in the fully adjusted model. However, the associations of continuous mean TG and TG/HDL-C levels, as well as the third and the top quartile of mean TG, became significant.

## Discussion

In this population-based cohort conducted in the MENA region with long-term follow-up and a high burden of dyslipidemia and CVD events, we demonstrated that long-term LV of TC, HDL-C, LDL-C, TG, non-HDL-C, TC/HDL-C, and TG/HDL-C by four different indicators (SD, CV, ARV, and VIM) was not consistently associated with an increased risk of CVD, especially when the effect of mean lipid levels was taken into account. However, mean levels of each lipid parameter excluding HDL-C after adjustment for well-known confounders and even their variability was significantly associated with higher risk; moreover, an inverse association was found for mean HDL-C.

Our findings regarding the association of mean levels of TC, TG, HDL-C, LDL-C, non-HDL-C, TC/HDL-C, and TG/HDL-C with CVD are generally in line with the current literature (27–33). We also found that the effect of mean lipid levels and TC/HDL-C variability on CVD risk was generally more pronounced in the non-elderly population; Brunner et al. (34) used data from Multinational Cardiovascular Risk Consortium across 19 countries, estimated the probability of CVD by the age of 75 years among 524,444 subjects and reported that a 50% reduction of non-HDL-C levels decreased the risk of first MACE by the age of 75 years; this study showed that the risk was significantly higher among younger people for the same increasing levels of non-HDL-C and that the earlier cholesterol concentrations were reduced, the risk reduction was greater. Recently, we found that high baseline levels of TC, LDL-C, and non-HDL-C among the Tehranian population without initial CVD, diabetes, and with a calculated atherosclerotic cardiovascular disease (ASCVD) risk < 5% were significantly associated with incident CVD events (35). In the current study, we extended the previous research by showing that mean levels of different lipid measures during 6 years were independently associated with incident CVD events even in the presence of lipid variability indices.

In the present study, higher LV was not consistently associated with increased CVD risk. All of these associations attenuated and became non-significant after further adjustment with mean lipid measurements. However, we found a non-linear relationship between VIM-TG/HDL-C and the risk of CVD, as those in the third quartile of VIM-TG/HDL-C had a 26% lower risk for the event. We also found that SD-TC/HDL-C was only associated with higher CVD risk among those aged < 60 years. The overall lack of a significant association between the variability of the lipid parameters and the risk of CVD was in contrast to previous studies. Recently, a systematic review and meta-analysis among 10 studies conducted in East Asia and the USA with follow-up durations ranging from 4.2 to 8.3 years found an increased CVD risk of 26–29% for TC variability, 11–18% for HDL-C, 9–16% for LDL-C, comparing top with bottom quartile; these associations were more robust for TC and HDL-C. Regarding TG, the association was inconclusive with significant heterogeneity between the included studies (9). Important sources of heterogeneity for this meta-analysis were attributable to the inclusion of the mean level of lipid parameters and using lipid-lowering medications as confounders in the multivariable-adjusted models. In our data analysis, in model 1, adjusted for a large set of covariates but not mean lipid levels, in both complete case and imputed data set, as a continuous variable, each 1-SD increase in TC and TC/HDL-C (SD and ARV) was associated with more than 10% increased risk of CVD events. However, no significant trend was found following categorizing data into quartiles.

In our study, during the 6-year measurement period, only 12% of the population reported taking lipid-lowering medications. The overall use of statins is low among the general Iranian population. In a nationwide survey among 21,293 Iranian adults in 2016, only 6.9% of the population were on statins (7). Of note, when we excluded statin users in our data set, no significant association was found between LV and CVD outcomes, even without adjustment with their related mean lipid levels. Park et al. (8), In a nationwide population-based cohort study of 1,934,324 statin-naive young adults, found that measures of lipid variability did not modify the risk of either MI or stroke; however, some studies found no effect modification in statin users for the effect of LV on cardiovascular outcomes (36, 37). However, the issue of drug compliance and adherence during the measurement period or follow-up and the initiation of lipid-lowering therapy was not addressed adequately in studies that found a higher risk for LV among the general population (38). Overall, we speculate that LV among Tehranian adults might be attributable to poor medication management and non-adherence to treatment, including lipid-lowering agents, rather than being truly representative of a causality.

Rodriguez et al. (39) showed that high adherence to drug treatment was associated with better survival among patients with ASCVD, as these individuals were less likely to have detrimental health behaviors, such as smoking or poor psychosocial support. Moreover, it is commonly cited that high variability in metabolic risk factors might be only a marker of increased risk, reflect general frailty, and be an epiphenomenon underlying unhealthy systemic conditions leading to adverse health outcomes (40). These controversial results between studies might also be due to the different population characteristics, lack of a standard LV parameter, duration of the measurement period, and the number of measurements. Additionally, although previous studies showed positive associations between LV and CVD, changes in the covariates were not taken into account (41).

### Strengths and limitations

Our study has several strengths, including the population-based and well-characterized design with a long-term follow-up conducted among Tehranian adults, which allowed us to study the effect of longitudinal measurements of all lipid parameters in a population with high burden of NCDs in the MENA region with direct administrative measurements instead of self-reported data. Furthermore, although previous studies mostly used covariates only from the index year, we considered the longitudinal measures of CVD risk factors (BMI, SBP, FPG, and 2-h PG) and initiated drug treatments over the 6-year measurement period to account for possible changes in the covariates and new cases of diabetes and hypertension. The following limitations merit consideration; first of all, although we controlled for a large set of well-known confounders, the possibility of residual confounding cannot be completely excluded. Second, lipid measurements were available at only 3 time points. It would be valuable to estimate variability with more visits occurring in the 6-year observational period. Third, the seasonal variations of serum lipid parameters were not accounted for in our study which might have led to an increased statistical noise. Fourth, LDL-C estimation by the Friedewald formula is inferior to direct LDL-C measurement, especially in the case of hypertriglyceridemia; however, we used the modified definition released by Chen et al. (13) that diminishes the interference caused by hypertriglyceridemia. Finally, our study population was from the metropolitan city of Tehran; hence, our results may not be generalizable to the country’s rural areas.

## Conclusion

In conclusion, from a clinical perspective, in a population with a high burden of NCDs, 6-year LV did not provide any information in predicting incident CVD events during more than a decade of follow-up. Focusing on the average lipid levels, TC, TG, HDL-C, LDL-C, TC-HDL-C, TG/HDL-C, and non-HDL-C, compared to their variability, are more informative in the prediction of incident CVD among the Iranian population. Our findings highlight the importance of achieving normal lipid levels over time, but not necessarily consistent, for averting adverse clinical outcomes.

## Data availability statement

The raw data supporting the conclusions of this article will be made available by the authors, without undue reservation.

## Ethics statement

The studies involving human participants were reviewed and approved by the Institutional Review Board (IRB) of the Research Institute for Endocrine Sciences (RIES), Shahid Beheshti University of Medical Sciences. The patients/participants provided their written informed consent to participate in this study.

## Author contributions

SM and FH contributed to the conception and design of the work. LC, SM, and NC contributed to the acquisition, analysis, and interpretation of data for the work. SM and ND drafted the manuscript. FH and MT critically revised the manuscript. All authors contributed to the article and approved the submitted version.
