# Supplementary material for: Mean versus variability of lipid measurements over 6 years and incident cardiovascular events: More than a decade follow-up
Source: Front Cardiovasc Med. 2022 Dec 9;9:1065528. doi: 10.3389/fcvm.2022.1065528 (PMC9780476; doi:10.3389/fcvm.2022.1065528)
Supplement: Supplementary file 1 [file Data_Sheet_1.docx]

| **Table S1.** Characteristics of the study population according to the incident CVD: Tehran Lipid and Glucose Study | | | | |
| --- | --- | --- | --- | --- |
|  | Total  (n = 3700) | No incident CVD  (n = 3351) | Incident CVD  (n = 349) | P-value |
| Baseline characteristics | | | | |
| Age (year) | 46.59 ± 11.39 | 45.76 ± 11.15 | 54.51 ± 10.64 | <0.01 |
| Baseline SBP (mmHg) | 117.11 ± 17.53 | 116.00 ± 16.75 | 127.74 ± 21.01 | <0.01 |
| SBP levels 2^nd^ exam | 116.99 ± 18.37 | 115.85 ± 17.86 | 127.88 ± 19.66 | <0.01 |
| SBP levels 3^rd^ exam | 120.07 ± 18.74 | 119.01 ± 18.23 | 130.16 ± 20.55 | <0.01 |
| Baseline BMI (kg/m^2^) | 28.11 ± 4.45 | 28.03 ± 4.45 | 28.88 ± 4.34 | <0.01 |
| BMI levels 2^nd^ exam | 28.38 ± 4.47 | 28.32 ± 4.47 | 28.99 ± 4.49 | 0.01 |
| BMI levels 3^rd^ exam | 28.87 ± 4.71 | 28.83 ± 4.70 | 29.19 ± 4.75 | 0.18 |
| Baseline FPG (mmol/L) | 5.48 ± 1.63 | 5.40 ± 1.50 | 6.30 ± 2.41 | <0.01 |
| Baseline 2-h PG (mmol/L) | 6.70 ± 3.01 | 6.59 ± 2.85 | 7.89 ± 4.26 | <0.01 |
| Male sex (%) | 1543 (41.7) | 1354 (40.4) | 189 (54.2) | <0.01 |
| Current smoking (%) | 428 (11.6) | 380 (11.3) | 48 (13.8) | 0.18 |
| Use of lipid-lowering drugs (%) | 466 (12.6) | 387 (11.5) | 79 (22.6) | <0.01 |
| Use of anti-hypertensive drugs (%) | 614 (16.6) | 498 (14.9) | 116 (33.2) | <0.01 |
| Hypertension | 644 (17.4) | 522 (15.6) | 122 (35.0) | <0.01 |
| Diabetes | 718 (19.4) | 586 (17.5) | 132 (37.8) | <0.01 |
| FH-CVD, yes | 375 (10.1) | 323 (9.6) | 52 (14.9) | <0.01 |
| Baseline lipid levels (mmol/L) | | | | |
| TC | 5.13 ± 1.02 | 5.10 ± 1.02 | 5.45 ± 1.05 | <0.01 |
| HDL-C | 1.00 ± 0.26 | 1.01 ± 0.26 | 0.95 ± 0.25 | <0.01 |
| LDL-C | 3.29 ± 0.83 | 3.26 ± 0.83 | 3.55 ± 0.85 | <0.01 |
| TG | 1.89 ±1.09 | 1.86 ± 1.08 | 2.18 ± 1.21 | <0.01 |
| non-HDL-C | 4.13 ± 1.02 | 4.10 ± 1.01 | 4.50 ± 1.02 | <0.01 |
| TC/HDL-C | 5.44 ± 1.66 | 5.37 ± 1.64 | 6.05 ± 1.71 | <0.01 |
| TG/HDL-C | 2.14 ± 1.64 | 2.09 ± 1.61 | 2.54 ± 1.79 | <0.01 |
| Lipid levels 2^nd^ exam (mmol/L) | | | | |
| TC | 5.11 ± 0.99 | 5.08 ± 0.98 | 5.37 ± 1.06 | <0.01 |
| HDL-C | 1.07 ± 0.26 | 1.08 ± 0.26 | 1.03 ± 0.24 | <0.01 |
| LDL-C | 3.20 ± 0.80 | 3.18 ± 0.79 | 3.42 ± 0.86 | <0.01 |
| TG | 1.89 ± 1.11 | 1.86 ± 1.10 | 2.08 ± 1.15 | <0.01 |
| non-HDL-C | 4.04 ± 0.98 | 4.01 ± 0.97 | 4.33 ± 1.04 | <0.01 |
| TC/HDL-C | 5.01 ± 1.14 | 4.97 ± 1.41 | 5.40 ± 1.40 | <0.01 |
| TG/HDL-C | 1.97 ± 1.56 | 1.94 ± 1.56 | 2.22 ± 1.56 | <0.01 |
| Lipid levels 3^rd^ exam (mmol/L) | | | | |
| TC | 5.15 ± 1.00 | 5.13 ± 0.98 | 5.37 ± 1.15 | <0.01 |
| HDL-C | 1.22 ± 0.29 | 1.23 ± 0.29 | 1.17 ± 0.26 | <0.01 |
| LDL-C | 3.13 ± 0.81 | 3.11 ± 0.79 | 3.32 ± 0.93 | <0.01 |
| TG | 1.79 ± 1.01 | 1.77 ± 0.99 | 1.99 ± 1.13 | <0.01 |
| non-HDL-C | 3.93 ± 0.99 | 3.90 ± 0.97 | 4.20 ± 1.11 | <0.01 |
| TC/HDL-C | 4.41 ± 1.26 | 4.38 ± 1.26 | 4.75 ± 1.22 | <0.01 |
| TG/HDL-C | 1.64 ± 1.26 | 1.61 ± 1.24 | 1.87 ± 1.41 | <0.01 |
| Abbreviations: CVD, cardiovascular disease; SBP, systolic blood pressure; BMI, body mass index; FPG, fasting plasma glucose; 2-h PG, 2 h post-challenge plasma glucose; FH-CVD, family history of CVD; TG, triglycerides; TC, total cholesterol; HDL-C, high-density lipoprotein cholesterol; LDL-C, low-density lipoprotein cholesterol. | | | | |
