# Supplementary material for: Mean versus variability of lipid measurements over 6 years and incident cardiovascular events: More than a decade follow-up
Source: Front Cardiovasc Med. 2022 Dec 9;9:1065528. doi: 10.3389/fcvm.2022.1065528 (PMC9780476; doi:10.3389/fcvm.2022.1065528)
Supplement: Supplementary file 2 [file Data_Sheet_2.docx]

| **Table S2.** HRs and 95% CIs of incident CVD for TC, TG, HDL-C, and LDL-C variability in the imputed dataset: Tehran Lipid and Glucose Study | | | | | | | | | | | | |
| --- | --- | --- | --- | --- | --- | --- | --- | --- | --- | --- | --- | --- |
|  | SD | | | CV | | | ARV | | | VIM | | |
|  | Model 1 | Model 2 | P trend | Model 1 | Model 2 | P trend | Model 1 | Model 2 | P trend | Model 1 | Model 2 | P  trend |
| TC | | | | | | | | | |  |  |  |
| Q_1_ | 1 (reference) | 1 (reference) | 0.72 | 1 (reference) | 1 (reference) | 0.94 | 1 (reference) | 1 (reference) | 0.38 | 1 (reference) | 1 (reference) | 0.98 |
| Q_2_ | 0.91 (0.69-1.21) | 0.88 (0.67-1.17) |  | 0.85 (0.64-1.12) | 0.84 (0.64-1.12) |  | 1.03 (0.78-1.37) | 1.00 (0.76-1.33) |  | 0.88 (0.67-1.16) | 0.88 (0.66-1.15) |  |
| Q_3_ | 1.02 (0.77-1.35) | 0.96 (0.73-1.28) |  | 0.94 (0.72-1.24) | 0.95 (0.72-1.25) |  | 1.13 (0.86-1.48) | 1.06 (0.81-1.40) |  | 0.95 (0.73-1.25) | 0.95 (0.73-1.25) |  |
| Q_4_ | 1.15 (0.87-1.52) | 1.02 (0.77-1.36) |  | 0.96 (0.72-1.26) | 0.98 (0.74-1.29) |  | 1.24 (0.94-1.64) | 1.12 (0.85-1.49) |  | 0.96 (0.73-1.27) | 0.97 (0.74-1.28) |  |
| Per 1-SD increase | 1.08 (1.00-1.17) | 1.01 (0.93-1.09) |  | 1.06 (0.97-1.16) | 1.05 (0.96-1.15) |  | 1.08 (1.00-1.17) | 1.01 (0.93-1.09) |  | 1.07 (0.97-1.17) | 1.05 (0.96-1.15) |  |
| TG | | | | | | | | | | | | |
| Q_1_ | 1 (reference) | 1 (reference) | 0.27 | 1 (reference) | 1 (reference) | 0.43 | 1 (reference) | 1 (reference) | 0.47 | 1 (reference) | 1 (reference) | 0.58 |
| Q_2_ | 1.07 (0.81-1.41) | 1.06 (0.80-1.40) |  | 0.98 (0.75-1.28) | 0.98 (0.75-1.28) |  | 1.04 (0.78-1.38) | 1.02 (0.77-1.36) |  | 0.88 (0.67-1.14) | 0.88 (0.67-1.15) |  |
| Q_3_ | 1.13 (0.85-1.51) | 1.10 (0.82-1.47) |  | 0.99 (0.76-1.29) | 0.98 (0.75-1.27) |  | 1.29 (0.97-1.71) | 1.24 (0.93-1.65) |  | 0.96 (0.74-1.25) | 0.96 (0.74-1.25) |  |
| Q_4_ | 1.29 (0.98-1.70) | 1.19 (0.87-1.64) |  | 1.16 (0.89-1.51) | 1.12 (0.86-1.46) |  | 1.18 (0.90-1.55) | 1.05 (0.76-1.44) |  | 1.07 (0.82-1.39) | 1.05 (0.81-1.38) |  |
| Per 1-SD increase | 1.06 (0.98-1.15) | 1.00 (0.90-1.13) |  | 1.03 (0.94-1.13) | 1.01 (0.92-1.12) |  | 1.03 (0.95-1.12) | 0.96 (0.86-1.08) |  | 1.01 (0.92-1.12) | 1.01 (0.91-1.11) |  |
| HDL-C | | | | | | | | | | | | |
| Q_1_ | 1 (reference) | 1 (reference) | 0.49 | 1 (reference) | 1 (reference) | 0.56 | 1 (reference) | 1 (reference) | 0.79 | 1 (reference) | 1 (reference) | 0.58 |
| Q_2_ | 1.23 (0.95-1.59) | 1.24 (0.96-1.61) |  | 0.93 (0.71-1.22) | 0.92 (0.70-1.21) |  | 0.93 (0.72-1.20) | 0.94 (0.73-1.22) |  | 0.98 (0.74-1.30) | 0.98 (0.74-1.29) |  |
| Q_3_ | 1.04 (0.79-1.37) | 1.08 (0.81-1.42) |  | 1.07 (0.82-1.40) | 1.07 (0.82-1.39) |  | 0.98 (0.75-1.28) | 1.01 (0.77-1.32) |  | 1.08 (0.83-1.42) | 1.08 (0.83-1.41) |  |
| Q_4_ | 1.06 (0.81-1.40) | 1.16 (0.87-1.54) |  | 1.04 (0.80-1.36) | 1.03 (0.79-1.35) |  | 0.87 (0.66-1.14) | 0.94 (0.71-1.24) |  | 1.05 (0.80-1.37) | 1.05 (0.80-1.37) |  |
| Per 1-SD increase | 1.00 (0.91-1.11) | 1.04 (0.94-1.16) |  | 1.06 (0.96-1.16) | 1.05 (0.96-1.16) |  | 0.96 (0.87-1.07) | 1.00 (0.89-1.11) |  | 1.05 (0.96-1.16) | 1.05 (1.04-1.16) |  |
| LDL-C | | | | | | | | | | | | |
| Q_1_ | 1 (reference) | 1 (reference) | 0.39 | 1 (reference) | 1 (reference) | 0.50 | 1 (reference) | 1 (reference) | 0.89 | 1 (reference) | 1 (reference) | 0.48 |
| Q_2_ | 1.04 (0.79-1.38) | 1.02 (0.77-1.35) |  | 0.89 (0.68-1.17) | 0.91 (0.69-1.19) |  | 0.98 (0.74-1.29) | 0.95 (0.72-1.27) |  | 0.93 (0.71-1.22) | 0.93 (0.71-1.23) |  |
| Q_3_ | 0.97 (0.73-1.28) | 0.91 (0.69-1.20) |  | 0.84 (0.64-1.10) | 0.86 (0.66-1.13) |  | 0.97 (0.73-1.29) | 0.92 (0.69-1.22) |  | 0.88 (0.67-1.15) | 0.88 (0.67-1.15) |  |
| Q_4_ | 1.04 (0.79-1.37) | 0.91 (0.69-1.21) |  | 0.85 (0.64-1.13) | 0.92 (0.70-1.22) |  | 1.15 (0.87-1.51) | 1.03 (0.78-1.36) |  | 0.91 (0.69-1.20) | 0.92 (0.70-1.22) |  |
| Per 1-SD increase | 1.05 (0.97-1.15) | 0.98 (0.90-1.07) |  | 0.99 (0.90-1.09) | 1.02 (0.93-1.12) |  | 1.07 (0.99-1.17) | 1.00 (0.92-1.09) |  | 1.02 (0.93-1.12) | 1.01 (0.92-1.10) |  |
| Model 1: Age, sex, smoking, lipid-lowering drugs, anti-hypertensive drugs, family history of CVD, diabetes, and averages of body mass index and systolic blood pressure.  Model 2: Model 1 + average lipid levels for each lipid parameter (TC, TG, HDL-C, LDL-C).  Abbreviations: HR, hazard ratio; CI, confidence interval; CVD, cardiovascular disease; TC, total cholesterol; TG, triglycerides; HDL-C, high-density lipoprotein cholesterol; LDL-C, low-density lipoprotein cholesterol; SD, standard deviation; CV, coefficient of variation; ARV, average real variability; VIM, variability independent of mean. | | | | | | | | | | | | |

| **Table S3.** HRs and 95% CIs of incident CVD for TC/HDL-C, TG/HDL-C, and non-HDL-C variability in the imputed dataset: Tehran Lipid and Glucose Study | | | | | | | | | | | | |
| --- | --- | --- | --- | --- | --- | --- | --- | --- | --- | --- | --- | --- |
|  | SD | | | CV | | | ARV | | | VIM | | |
|  | Model 1 | Model 2 | P trend | Model 1 | Model 2 | P trend | Model 1 | Model 2 | P trend | Model 1 | Model 2 | P trend |
| TC/HDL-C | | | | | | | | | | | | |
| Q_1_ | 1 (reference) | 1 (reference) | 0.99 | 1 (reference) | 1 (reference) | 0.83 | 1 (reference) | 1 (reference) | 0.71 | 1 (reference) | 1 (reference) | 0.99 |
| Q_2_ | 0.91 (0.67-1.23) | 0.87 (0.64-1.17) |  | 0.97 (0.72-1.30) | 0.94 (0.70-1.26) |  | 0.87 (0.64-1.17) | 0.83 (0.61-1.11) |  | 0.90 (0.67-1.21) | 0.87 (0.65-1.17) |  |
| Q_3_ | 1.01 (0.76-1.34) | 0.91 (0.68-1.21) |  | 0.96 (0.73-1.27) | 0.93 (0.70-1.23) |  | 1.07 (0.81-1.42) | 0.96 (0.72-1.27) |  | 0.89 (0.68-1.18) | 0.89 (0.67-1.17) |  |
| Q_4_ | 1.25 (0.96-1.63) | 0.98 (0.73-1.30) |  | 1.09 (0.83-1.43) | 1.03 (0.78-1.35) |  | 1.26 (0.97-1.65) | 1.00 (0.75-1.33) |  | 0.98 (0.75-1.29) | 0.99 (0.75-1.30) |  |
| Per 1-SD increase | 1.12 (1.04-1.21) | 1.02 (0.93-1.11) |  | 1.05 (0.96-1.15) | 1.02 (0.93-1.12) |  | 1.12 (1.04-1.22) | 1.02 (0.93-1.12) |  | 1.01 (0.92-1.11) | 1.02 (0.93-1.12) |  |
| TG/HDL-C | | | | | | | | | | | | |
| Q_1_ | 1 (reference) | 1 (reference) | 0.56 | 1 (reference) | 1 (reference) | 0.98 | 1 (reference) | 1 (reference) | 0.89 | 1 (reference) | 1 (reference) | 0.94 |
| Q_2_ | 0.97 (0.72-1.31) | 0.96 (0.71-1.29) |  | 0.89 (0.68-1.16) | 0.88 (0.67-1.15) |  | 0.90 (0.68-1.20) | 0.88 (0.66-1.17) |  | 0.84 (0.64-1.10) | 0.84 (0.64-1.10) |  |
| Q_3_ | 0.99 (0.75-1.31) | 0.96 (0.72-1.28) |  | 0.82 (0.62-1.08) | 0.81 (0.62-1.07) |  | 1.02 (0.77-1.35) | 0.97 (0.73-1.29) |  | 0.81 (0.62-1.06) | 0.81 (0.62-1.06) |  |
| Q_4_ | 1.22 (0.93-1.60) | 1.12 (0.80-1.56) |  | 1.07 (0.83-1.37) | 1.03 (0.80-1.33) |  | 1.08 (0.83-1.42) | 0.94 (0.68-1.31) |  | 1.04 (0.80-1.34) | 1.02 (0.79-1.32) |  |
| Per 1-SD increase | 1.05 (0.97-1.13) | 0.97 (0.86-1.10) |  | 1.01 (0.92-1.11) | 1.00 (0.91-1.10) |  | 1.03 (0.95-1.12) | 0.93 (0.82-1.06) |  | 0.99 (0.90-1.09) | 0.99 (0.90-1.09) |  |
| Non-HDL-C | | | | | | | | | | | | |
| Q_1_ | 1 (reference) | 1 (reference) | 0.33 | 1 (reference) | 1 (reference) | 0.90 | 1 (reference) | 1 (reference) | 0.82 | 1 (reference) | 1 (reference) | 0.72 |
| Q_2_ | 0.93 (0.71-1.22) | 0.91 (0.70-1.20) |  | 0.93 (0.72-1.21) | 0.94 (0.72-1.23) |  | 0.82 (0.62-1.09) | 0.80 (0.61-1.07) |  | 0.95 (0.72-1.24) | 0.95 (0.72-1.24) |  |
| Q_3_ | 0.88 (0.67-1.16) | 0.84 (0.63-1.10) |  | 0.86 (0.66-1.14) | 0.89 (0.68-1.18) |  | 0.97 (0.74-1.27) | 0.91 (0.69-1.20) |  | 0.87 (0.66-1.14) | 0.87 (0.66-1.14) |  |
| Q_4_ | 1.00 (0.76-1.32) | 0.89 (0.67-1.18) |  | 0.94 (0.71-1.24) | 1.00 (0.76-1.32) |  | 1.09 (0.83-1.44) | 0.99 (0.75-1.30) |  | 0.96 (0.73-1.27) | 0.97 (0.74-1.28) |  |
| Per 1-SD increase | 1.05 (0.97-1.15) | 0.98 (0.90-1.06) |  | 1.00 (0.91-1.10) | 1.02 (0.93-1.12) |  | 1.07 (0.99-1.17) | 0.99 (0.91-1.08) |  | 1.02 (0.93-1.13) | 1.01 (0.92-1.10) |  |
| Model 1: Age, sex, smoking, lipid-lowering drugs, anti-hypertensive drugs, family history of CVD, diabetes, and averages of body mass index and systolic blood pressure.  Model 2: Model 1 + average lipid levels for each lipid parameter (TC/HDL-C, TG/HDL-C, non-HDL-C).  Abbreviations: HR, hazard ratio; CI, confidence interval; CVD, cardiovascular disease; SD, standard deviation; CV, coefficient of variation; ARV, average real variability; VIM, variability independent of mean; TC, total cholesterol; TG, triglycerides; HDL-C, high-density lipoprotein cholesterol. | | | | | | | | | | | | |

**Table S4.** HRs and 95% CIs of incident CVD for the mean of lipid parameters in the imputed dataset: Tehran Lipid and Glucose Study

|  | HR (95%CI) | P trend |  |  | HR (95%CI) | P trend |
| --- | --- | --- | --- | --- | --- | --- |
| TC | | |  | TC/HDL-C |  |  |
| Q1 | 1 (reference) | <0.01 |  | Q1 | 1 (reference) | <0.01 |
| Q2 | 1.17 (0.86-1.59) |  |  | Q2 | 1.31 (0.95-1.82) |  |
| Q3 | 1.16 (0.86-1.56) |  |  | Q3 | 1.57 (1.15-2.15) |  |
| Q4 | 1.90 (1.44-2.49) |  |  | Q4 | 2.01 (1.49-2.70) |  |
| Per 1-SD increase | 1.24 (1.15-1.35) |  |  | Per 1-SD increase | 1.27 (1.16-1.38) |  |
| TG | | |  | TG/HDL-C |  |  |
| Q1 | 1 (reference) | 0.02 |  | Q1 | 1 (reference) | <0.01 |
| Q2 | 1.13 (0.83-1.54) |  |  | Q2 | 1.24 (0.90-1.70) |  |
| Q3 | 1.40 (1.04-1.88) |  |  | Q3 | 1.57 (1.17-2.11) |  |
| Q4 | 1.38 (1.02-1.85) |  |  | Q4 | 1.46 (1.08-1.97) |  |
| Per 1-SD increase | 1.09 (1.00-1.18) |  |  | Per 1-SD increase | 1.09 (1.00-1.18) |  |
| HDL-C | | |  | Non-HDL-C |  |  |
| Q1 | 1 (reference) | 0.04 |  | Q1 | 1 (reference) | <0.01 |
| Q2 | 0.93 (0.73-1.19) |  |  | Q2 | 1.22 (0.89-1.69) |  |
| Q3 | 0.90 (0.69-1.16) |  |  | Q3 | 1.35 (0.99-1.83) |  |
| Q4 | 0.74 (0.56-0.98) |  |  | Q4 | 2.11 (1.59-2.81) |  |
| Per 1-SD increase | 0.89 (0.81-0.99) |  |  | Per 1-SD increase | 1.27 (1.17-1.37) |  |
| LDL-C | | |  | | | |
| Q1 | 1 (reference) | <0.01 |  |  |  |  |
| Q2 | 1.16 (0.85-1.60) |  |  |  |  |  |
| Q3 | 1.25 (0.93-1.69) |  |  |  |  |  |
| Q4 | 1.97 (1.49-2.59) |  |  |  |  |  |
| Per 1-SD increase | 1.28 (1.18-1.38) |  |  |  |  |  |
| Age, sex, smoking, lipid-lowering drugs, anti-hypertensive drugs, family history of CVD, diabetes, and averages of body mass index and systolic blood pressure.  Abbreviations: HR, hazard ratio; CI, confidence interval; CVD, cardiovascular disease; SD, standard deviation; CV, coefficient of variation; ARV, average real variability; VIM, variability independent of mean; TC, total cholesterol; Q, quartile; TG, triglycerides; HDL-C, high-density lipoprotein cholesterol; LDL-C, low-density lipoprotein cholesterol. | | | | | | |
