# Supplementary material for: Mean versus variability of lipid measurements over 6 years and incident cardiovascular events: More than a decade follow-up
Source: Front Cardiovasc Med. 2022 Dec 9;9:1065528. doi: 10.3389/fcvm.2022.1065528 (PMC9780476; doi:10.3389/fcvm.2022.1065528)
Supplement: Supplementary file 3 [file Data_Sheet_3.docx]

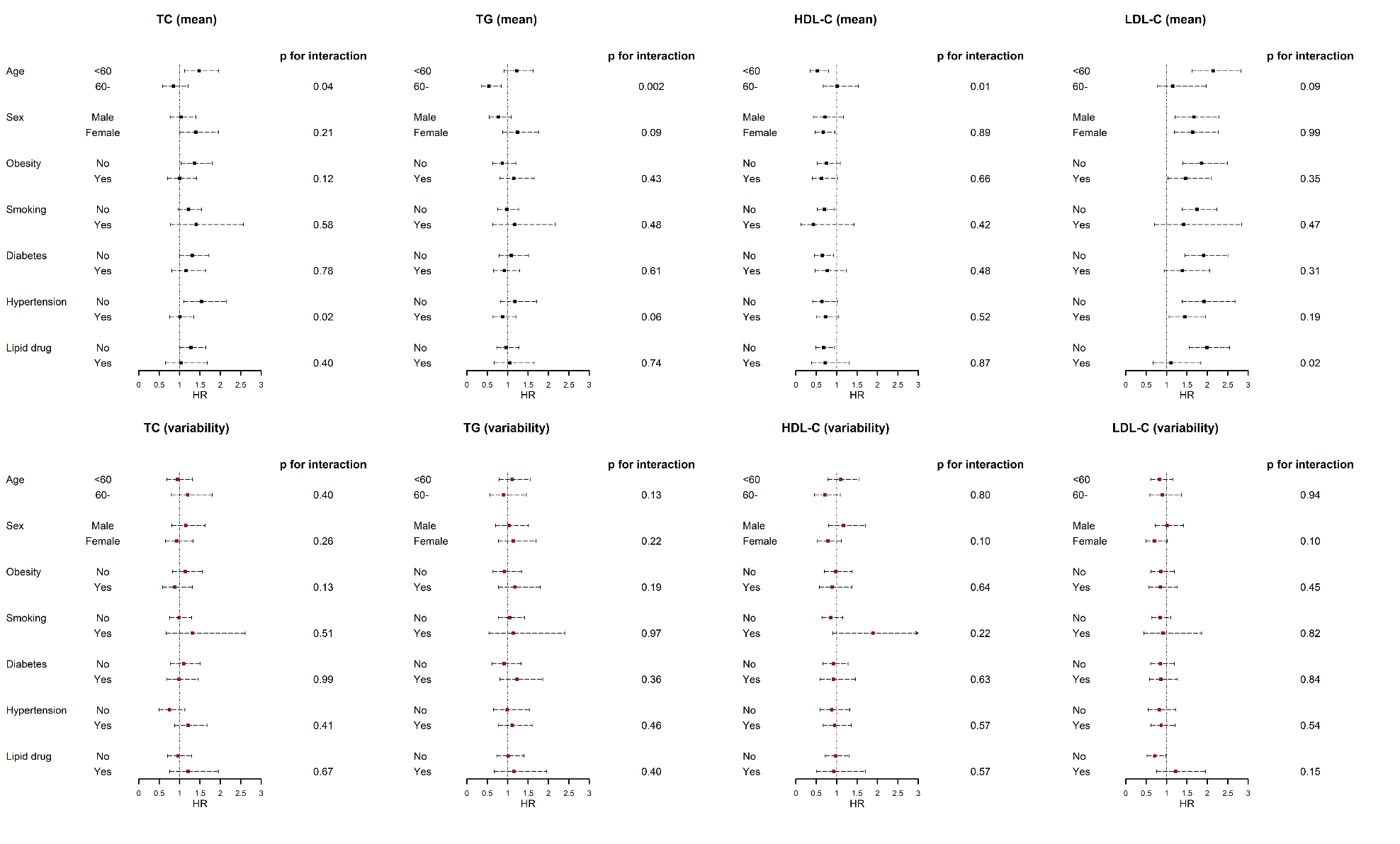
**Figure S1.** Multivariable-adjusted HRs (95% CI) of CVD for TC, TG, HDL-C, and LDL-C variability and mean levels in subgroups (Q4 versus Q1-3). Adjusted for age, sex, smoking, lipid-lowering drugs, anti-hypertensive drugs, family history of CVD, diabetes, and average BMI and SBP values; for the variability analyses, further adjusted for mean lipid levels. Each subgroup was assessed independently. HR; hazard ratio, CI; confidence interval, TC; total cholesterol, TG; triglycerides, HDL-C; high-density lipoprotein cholesterol, LDL-C; low-density lipoprotein cholesterol.


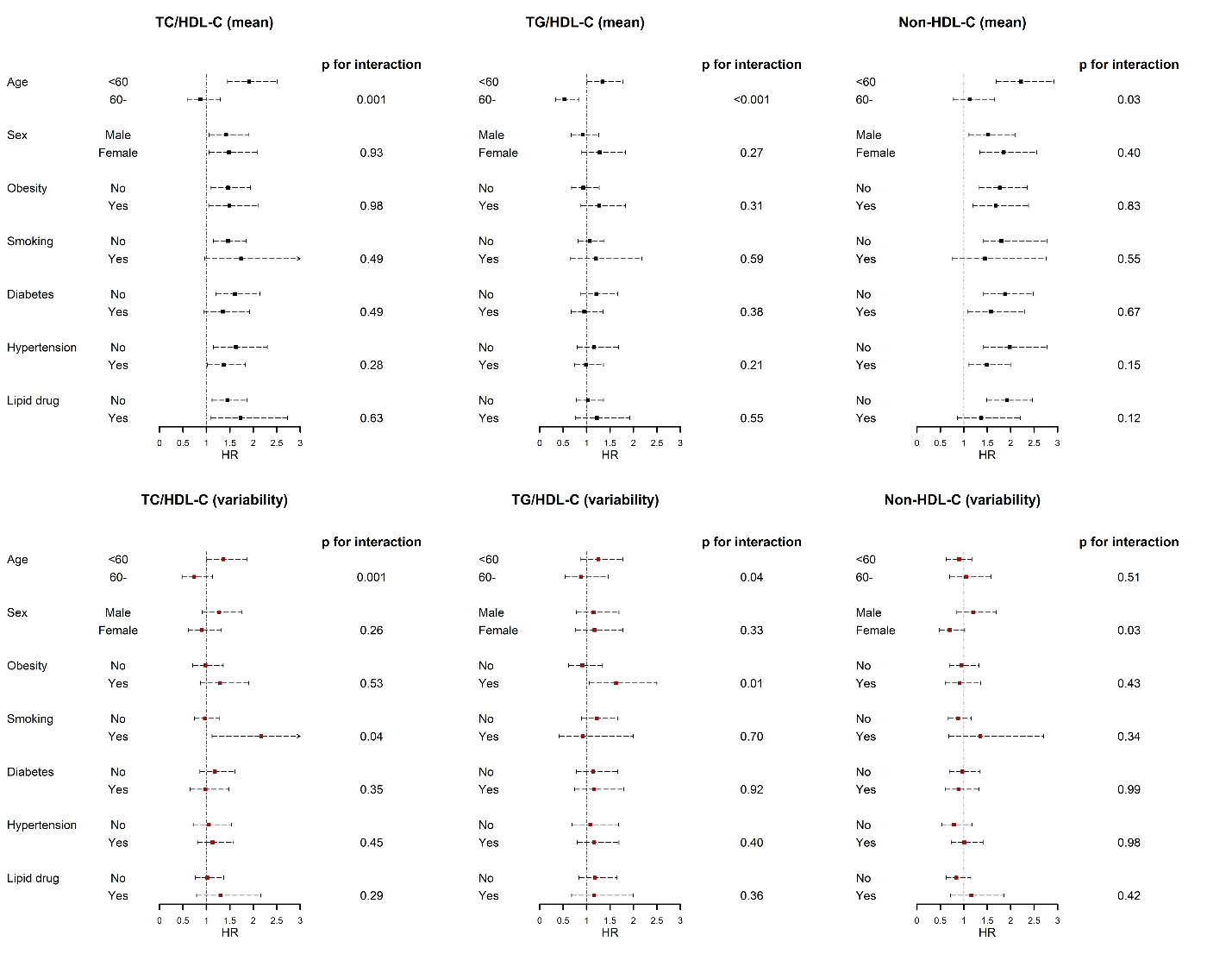


**Figure S2.** Multivariable-adjusted HRs (95% CI) of CVD for TC/HDL-C, TG/HDL-C, and non-HDL-C variability and mean levels in subgroups (Q4 versus Q1-3). Adjusted for age, sex, smoking, lipid-lowering drugs, anti-hypertensive drugs, family history of CVD, diabetes, and average BMI and SBP values; for the variability analyses, further adjusted for mean lipid levels. Each subgroup was assessed independently. HR; hazard ratio, CI; confidence interval, TC; total cholesterol, TG; triglycerides, HDL-C; high-density lipoprotein cholesterol.
